# Analysis of Tangential Leakage Flow Characteristics of Oil-Free Scroll Expander for a Micro-Scale Compressed Air Energy Storage System

**DOI:** 10.3390/e25020339

**Published:** 2023-02-12

**Authors:** Jian Sun, Bin Peng, Bingguo Zhu, Yaohong Li

**Affiliations:** School of Mechanical & Electronical Engineering, Lanzhou University of Technology, Lanzhou 730050, China

**Keywords:** micro-scale compressed air energy storage system, scroll expander, computational fluid dynamics, numerical simulation, tangential leakage

## Abstract

Tangential leakage loss is the primary factor that significantly affects the output performance of oil-free scroll expanders. A scroll expander can function under different operating conditions, and the flow of tangential leakage and generation mechanism is different. This study employed computational fluid dynamics to investigate the unsteady flow characteristics of the tangential leakage flow of a scroll expander with air as the working fluid. Consequently, the effects of different radial gap sizes, rotational speeds, inlet pressures, and temperatures on the tangential leakage were discussed. The tangential leakage decreased with increases in the scroll expander rotational speed, inlet pressure, and temperature, and decreased with decrease in radial clearance. With an equal-proportional increase in radial clearance, the flow form of the gas in the first expansion and back-pressure chambers became more complicated; when the radial clearance increased from 0.2 to 0.5 mm, the volumetric efficiency of the scroll expander decreased by approximately 5.0521%. Moreover, because of the large radial clearance, the tangential leakage flow maintained a subsonic flow. Further, the tangential leakage decreased with increase in rotational speed, and when the rotational speed increased from 2000 to 5000 r/min, the volumetric efficiency increased by approximately 8.7565%.

## 1. Introduction

Compressed air energy storage (CAES) systems are crucial to addressing the storage and release of electricity from renewable sources such as solar and photovoltaic power, and are in their initial commercialization stage worldwide [1]. A compressed-air energy storage system mainly consists of compressed air system, gas storage system, expansion-generation system, auxiliary system, and control system. When the electricity price is low, the low temperature and low-pressure air is compressed by the compressor into high pressure gas and stored in the gas storage system; when the electricity is insufficient, the internal energy-mechanical energy-electricity conversion is realized by the expansion-generation system [2]. The compressor and expander are used in the compressed air system and the expansion-generation system, respectively. The oil-free scroll expander is a new type of volumetric scroll machine that can maintain a high volumetric efficiency during operation, rendering it suitable for Micro-Scale CAES systems [3] and scale organic Rankine cycle (ORC) systems [4]. In contrast to oiled scroll machines, oil-free scroll expanders reduce the cost of oil–gas separation and thus improve the economy of waste heat utilization in the entire working system. However, owing to the existence of radial and axial clearances in the scroll expander structure, which cannot be avoided and eliminated during operation, the isentropic and volumetric efficiency of the scroll machine is reduced [5]. For scroll machines, both radial and axial clearances are the main factors affecting the volumetric and isentropic efficiency, whereas the internal leakage of scroll machines is primarily due to the operating conditions, machining, and assembly accuracy [6].

The construction of a model of internal leakage of scroll machinery is the main method used to study the amount of internal leakage between adjacent working chambers and its influence on the working process of scroll machinery. Choosing a suitable method for constructing an internal leakage model is important for improving the accuracy of the mathematical model calculation [7]. Fadhel et al. [8] constructed a mathematical model considering internal leakage and heat transfer for a fully closed scroll expander using the actual working fluid R245fa and verified the accuracy of the constructed thermodynamic model through experiments. They found that the inlet and outlet pressure significantly influenced the basic output performance of the expander. Emhardt et al. [9] studied the effect of radial gap size and pressure ratios on the output performance of a scroll expander. They reported that reduction in the radial gap size reduced the tangential leakage, resulting in significant increases in the isentropic efficiency and specific power of the scroll expander. Moreover, maximum efficiency was achieved when the scroll expander was in the isentropic compression state. Fanti et al. [10] designed two prototypes to control the radial clearance between the fixed and orbiting scroll by adjusting the eccentricity of the small crank, and built an experimental test platform for scroll expanders with air as the working fluid. They conducted experimental studies on two prototypes with different radial clearances to study the effect of tangential leakage on the performance of scroll expanders. Oh et al. [11] reported that using deterministic methods to construct mathematical models of scroll expanders was not suitable as the influence of factors such as geometric parameters and machining accuracy on the mathematical models could not be incorporated. Moreover, the results and conclusions obtained did not provide an in-depth analysis of the working process of the gas in the working chamber. Thus, they proposed a new method to establish a mathematical model that could comprehensively consider the influence of internal leakage and heat transfer on the expansion process. This mathematical model comprehensively reflected the variation of the working process in different working chambers of the scroll expander, and the errors of both experimental and simulation results were within 5%. As there are many uncertainties in the isentropic flow model used to predict the internal leakage of scroll compressors, Pereira et al. [12] constructed an internal leakage model considering refrigerant type, geometric parameters, and operating conditions. In addition, they proposed certain dimensionless parameters to evaluate the effect of internal leakage on scroll compressors. The constructed model more accurately reflected the actual operation of the compressor compared with the existing models for predicting internal leakage. Wang et al. [13] proposed an improved Fano flow and turbulence model to address the shortcomings of existing models for predicting radial leakage in scroll compressors. They proposed a simplified air compressor test bench to investigate the effects of differential pressure, axial clearance, and rotational speed on radial leakage. The Fano flow model can predict the radial leakage more accurately at low speed and small clearance (n=3500 r/min, $\delta_{a}=$0.025 mm). Moreover, at the same axial clearance, the radial leakage of compressor increased with increase in speed and differential pressure. Although the changing characteristics of the internal leakage of the scroll machinery can be predicted more accurately by constructing a mathematical model considering the leakage, it cannot truly reflect the specific effect of the internal leakage on the fluid flow in the working chamber, particularly the changing law of the fluid flow in the leakage gap [14].

For volumetric machinery, because the working chamber is closed, it is impossible to directly study and analyze the movement law of gas in the working chamber by direct means and methods [15]. CFD-based visualization studies facilitate a more intuitive examination and analysis of the flow law of fluid in the leakage gap and provide a simpler and more convenient method for reducing the leakage gap [16]. Sun et al. [17] conducted a three-dimensional unsteady-state numerical simulation of a scroll compressor considering radial and axial gaps using PumpLinx software based on CFD method. The flow form of the gas through the radial and axial gaps was found to be inconsistent, and the flow of the gas through the radial gap was dominated by the turbulent form. Further, the gas was susceptible to the supersonic flow form when subjected to different pressure ratios in the meshing gap. Zheng et al. [18] studied the formation characteristics and development mechanism of tangential leakage flow in different working chambers by considering the effects of radial clearance and roughness of scroll tooth on tangential leakage. The formation and flow mechanism of tangential leakage flow were found to differ in different working chambers owing to the mass exchange between adjacent working chambers causing the formation of secondary flow and passage vortex in the compression chamber. Consequently, the secondary flow increased the local temperature in the compressor working chamber and increase in the radial clearance significantly reduced the volumetric and isentropic efficiency of the compressor. Zha et al. [19] proposed a radial and tangential leakage model of a real gas considering both friction and compressibility, and conducted a three-dimensional steady-state numerical simulation study of the leakage model based on CFD method. The roughness of the scroll tooth wall surface exerted a greater impact on the internal leakage volume under the same leakage gap, and the leakage volume decreased with increase in the pressure difference. Rak et al. [20] proposed a new structured meshing method for the fluid domain to improve the mesh quality at the radial gap of a scroll compressor, and performed a three-dimensional unsteady numerical simulation of the compressor fluid domain based on CFD methods. The leakage gap was observed to affect the distribution of the flow field near the gap, and the average pressure inside the gap was proportional to the gap size. Sun et al. [21] analyzed a three-dimensional unsteady state numerical simulation of a scroll compressor using the actual working fluid R22 at a constant radial gap ($\delta_{r}=$0.03 mm), and the computational fluid domain was composed of a hexahedral structured mesh to improve the computational accuracy. The temperature and pressure in the working chamber tended to decrease and then increase when the fluid flowed through the radial gap. Zhang et al. [22] performed a three-dimensional unsteady state numerical simulation of a fluid domain considering both radial and tangential leakage based on the CFD method to improve the volumetric efficiency of a scroll hydrogen pump applied in a fuel cell. The leakage flowing through the axial gap was found to be higher than that generated by the radial gap for the same leakage gap size. Further, the smaller the leakage gap, the higher the volumetric efficiency of the compressor. Fadiga et al. [23] proposed a new structured meshing method for the fluid domain using the OpenFoam software to make the numerical simulation results of the scroll expander closer to the actual values. Consequently, the number of meshes and nodes were reduced, time required for meshing the fluid domain was shortened, and computational resources were saved. Zheng et al. [24] conducted the first three-dimensional unsteady-state numerical simulation of the working process of a transcritical CO_2_ scroll compressor to study and analyze the fluid distribution law in the working chamber. They also examined the effect of different compression processes on the fluid distribution law in the compression chamber. The uneven distribution of temperature and pressure in the compression chamber and the asymmetric arrangement of the compressor outlet resulted in a “quasi-centrosymmetric” leakage flow in the compression chamber. This phenomenon has rarely been studied in other materials. The oil-free double-wrap scroll compressor is suitable for large displacement and low-pressure ratio owing to its large discharge volume and low discharge pressure. Sun et al. [25] conducted unsteady state numerical simulations based on the CFD method for an scroll compressor applied to a hydrogen fuel cell, and studied the distribution law of the gas in the working chamber of the compressor as well as the inlet and outlet mass flow and velocity with the spindle rotation angle. Sun et al. [26] used the κ−ε RNK turbulence model to study the distribution law of fluid pressure, temperature, and velocity inside the working chamber of an oil-free air scroll compressor. The tangential leakage flow generated by the radial gap disturbed the distribution law of the gas inside the low-pressure chamber and exerted the greatest effect on the temperature and velocity of the gas inside the chamber.

The passive flow control method can control fluid flow in advance by setting up a passive device [27]. The labyrinth seal method has been fully studied and applied in other volumetric machines. Although it has also been studied in scroll machines, only a brief introduction has been made and the use and theory of the method has not been comprehensively studied and analyzed. Liu et al. [28] first applied the labyrinth seal method to the axial gap seal structure of a scroll compressor and constructed a radial leakage model of the compressor based on the energy and mass equations. The tangential leakage was reduced by approximately 21% by opening radial labyrinth seal grooves at the top of the orbiting and fixed scroll teeth compared with the smooth tooth top surface. Zheng et al. [29] established a CFD model of a radial labyrinth and studied the effect of parameters such as the number, width, and depth of the grooves on the radial leakage. They found that the radial leakage decreased with the increase in groove depth because the opened grooves produced a continuous throttling effect on the fluid, jet expansion, and energy flow dissipation. Therefore, opening grooves on the top of the scroll teeth can significantly reduce the losses caused by radial leakage. Further, Zheng et al. [30] opened sealing slots on the wall surface of the fixed and orbiting scroll teeth, and used numerical simulations to study the flow mechanism of the actual gas CO_2_ in the radial gap and the sealing slots in depth. The geometric parameters of the sealing slots were reported to exert a certain influence on the sealing effect, and increasing the number of sealing slots improved the volumetric and isentropic efficiency of the compressor.

The existing scroll mechanical internal leakage research is focused on the scroll compressor radial and tangential leakage research, whereas studies on the formation of internal leakage in the scroll expander and the mechanism of generation are scarce. Although the basic construction and operating principles of scroll compressors and expanders are similar, the flow mechanisms of fluids in the working chamber are different. Therefore, to study the effect of internal leakage on the performance of scroll expanders, a detailed study and analysis of the generation and flow mechanisms of internal leakage flows in expanders is required. Radial leakage caused by axial clearance can be eliminated via the addition of seals to the top of the fixed and orbiting scroll teeth; however, the tangential leakage caused by radial clearance cannot be eliminated. Therefore, a detailed analysis and study of the generation and flow mechanism of tangential leakage between adjacent working chambers of the scroll expander is required to reduce the losses caused by it and thus improve the work efficiency of the scroll expander. This study investigated the effects of radial gap size, rotational speed, inlet pressure, and temperature on the tangential leakage flow through three-dimensional unsteady state numerical simulations of an oil-free scroll expander. This study provides a certain theoretical basis and guidance for improving the basic output performance of scroll expanders under variable operating conditions.

## 2. Geometric Model and Leakage Model

### 2.1. Geometric Model

**Figure 1:**
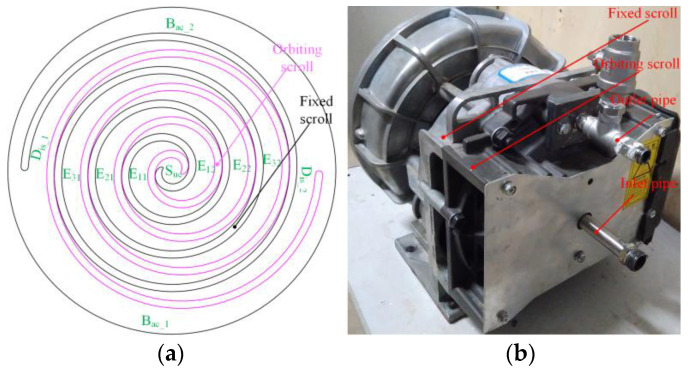
Scroll expander (**a**) Chamber division (**b**) Solid structure.

Figure 1a shows a schematic of the division of the working chamber of the scroll expander used at the institute. At this point the scroll expander has just completed a suction process, defined as the moment of initial movement of the expander (noted as θ= 0°). Where, S_uc_ is the suction chamber, D_is_ is the discharge chamber, B_ac_ is the back-pressure chamber, and E is the expansion chamber. Further, the different expansion chambers of the scroll expander are denoted by E*_ij_*, where *i* is the number of expansion chambers (*I* = 1, 2, 3) and *j* is the number of expansion chambers of the same name (*j* = 1, 2). The basic geometrical parameters of the scroll tooth profile are shown below: radius of base circle $r_{b}$ = 3.676 mm, involute angle α = 0.680 rad, and scroll tooth height *h* = 25 mm [31].

### 2.2. Tangential Leakage Model

**Figure 2:**
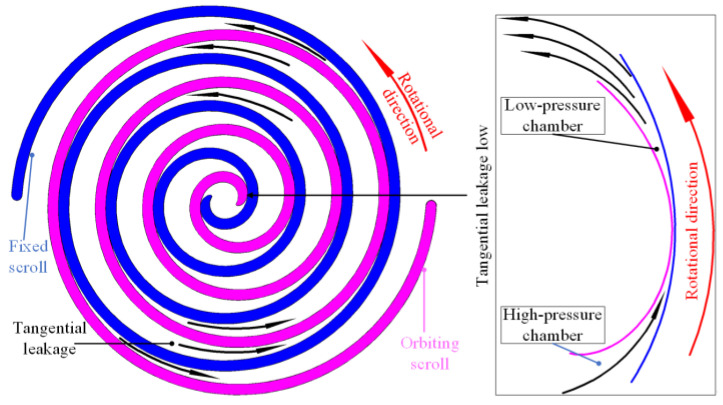
Schematic of tangential leakage.

A schematic of the tangential leakage of the scroll expander is shown in Figure 2. The tangential leakage of scroll machinery was generated by the meshing gap between the orbiting and fixed scroll teeth, which is referred to as the radial gap. Between adjacent working chambers a tangential leak will occur through the radial gap from the high-pressure chamber to the low-pressure chamber.

## 3. Numerical Simulation

### 3.1. Meshing

**Figure 3:**
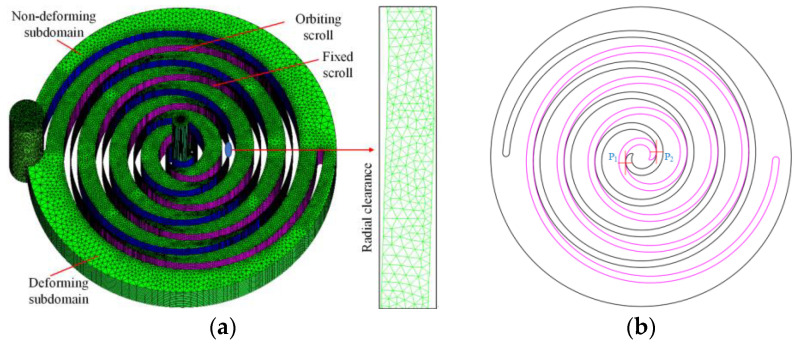
Scroll expander fluid domain grid (**a**) Fluid domain grid and radial clearance (**b**) Location of monitoring points.

Figure 3a shows a schematic of the meshing of the scroll expander fluid domain, which is divided into the suction and discharge regions and the working chamber region. To fit the divided mesh to the different fluid domains, hexahedral structured and trigonal unstructured meshes were used to form the inlet and outlet pipes and the working chamber region, respectively. As a sufficient number of meshes could not be drawn in the theoretical radial gap ($\delta_{r}=$ 0.02 mm) using unstructured meshes in ICEM, the meshing gap was enlarged by a factor of 10 (that is $\delta_{r}=$ 0.2 mm) to analyze and study the three-dimensional unsteady state numerical simulation of the scroll expander [32]. Figure 3b shows the arrangement of the monitoring points P_1_ and P_2_. In this case, P_1_ and P_2_ are located at the engagement points of the orbiting and fixed scroll teeth in the suction and the first expansion chambers E_11_ and E_12_, respectively, with both P_1_ and P_2_ being close to the fixed scroll teeth.

### 3.2. Control Equations and Inlet and Outlet Boundary Conditions

#### 3.2.1. Control Equations

For scroll machines, the RNG turbulence model is frequently used for three-dimensional unsteady state numerical simulations in the fluid domain. Owing to the working fluid used being air, the physical properties of air can be disregarded. The PISO! algorithm with good pressure-velocity coupling is used, and the time step was set to $10^{-5}$ s. Other computational parameters were set according to those found in the literature [24,33].

For the air scroll expander, considering the viscosity and compressibility of the gas in the working chamber, the Reynolds time averaged N-S equation was used to express the gas mass and momentum equation in the working chamber:

(1) $$\frac{\partial (\delta \psi )}{\partial d}+div(\delta \mu \psi )=div(D\cdot grad\psi )+S$$

(2) $$\frac{\partial (\delta \psi )}{\partial \zeta }+\frac{\partial (\delta \mu \psi )}{\partial x}+\frac{\partial (\nu \psi )}{\partial y}+\frac{\partial (\chi \psi )}{\partial z}=\frac{\partial }{\partial x}(D\frac{\partial \psi }{\partial x})+\frac{\partial }{\partial y}(D\frac{\partial \psi }{\partial y})+\frac{\partial }{\partial z}(D\frac{\partial \psi }{\partial z})+S$$

#### 3.2.2. Inlet and Outlet Boundary Conditions

According to the experimental tests and thermodynamic model, the rated operating conditions of the scroll expander were set as follows: the inlet and outlet pressures were $p_{s}$ = 900 KPa and $p_{d}$ = 200 KPa respectively, the inlet temperature was $T_{s}$ = 420.15 K, the outlet temperature was set as an open boundary condition, and the expander rotational speed was *n* = 3000 r/min.

### 3.3. Volumetric Efficiency and Grid-Independence Validation

#### 3.3.1. Volumetric Efficiency

The volumetric efficiency reflects the usage efficiency of the inner volume of the scroll machinery and can be used to measure the degree of internal leakage within the working chamber of the scroll expander. This is calculated as follows:

(3) $$\eta_{v}=\frac{m_{\mathrm{sim}}}{n\cdot V_{s}\cdot \rho_{s}}$$

#### 3.3.2. Grid-Independent Verification

To conduct a grid-independent verification, the rated operating conditions set in Section 3.2.2 were chosen to perform numerical simulations for different numbers of fluid domain grids. Table 1 presents the volumetric efficiency of the scroll expander and the computational time required for one operating cycle for different number of fluid domain grids. To save on computational resources and time, the number of fluid domain grids was set as 2,858,032 for the numerical simulation.

The volumetric and isentropic efficiencies of the scroll expander at rated operating conditions are shown in Table 2. Since the radial clearance was enlarged by a factor of 10 when the numerical simulation was performed, this would make the numerical simulation values lower than the experimental values. Since the error between the numerical simulation and the experimental values of the volumetric efficiency and isentropic efficiency is less than 15%, it can be shown that the CFD model can reflect the actual working process of the scroll expander more accurately.

**Figure 4:**
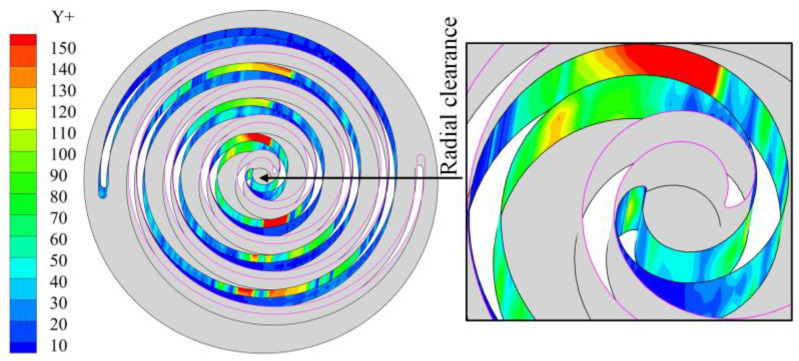
Y+ distribution on the wall surface of the fixed scroll.

Figure 4 shows the contour distribution of the wall Y+ of the fixed scroll teeth of the scroll expander. As is evident, the wall Y+ values above 100 are located in the working chamber, the velocity in the chamber is not very large, and the wall Y+ values at the meshing gap of the fixed scroll teeth are less than 50. Thus, the number of meshes selected do not exert a significant influence on the calculation results [17,24].

## 4. Results and Analysis

When analyzing the results of a three-dimensional unsteady state numerical simulation of a scroll expander, it was considered that the expander reached dynamic equilibrium during its motion when the difference between the inlet and outlet mass flow was within 1% of each other in a cycle [24,34]. To study the effect of radial clearances size on the tangential leakage of the scroll expander, only the radial gap size was varied, without changing other calculated initial conditions. Further, the effect of rotational speed on tangential leakage was investigated by varying the user defined function (UDF) time term.

For scroll machines, the tangential leakage between adjacent working chambers was owing to the movement of gas from the high-pressure chamber to the low-pressure chamber through the radial clearance. Existing research data indicate that the internal leakage between the central suction and adjacent working chamber is the largest, that is, the tangential leakage between the first expansion chamber E_1*j*_ and the suction chamber S_uc_ is much higher than that between the other working chambers [10,30].

Assuming that the flow state of the gas as it passes through the radial gap is laminar, the following equation for the Reynolds number of the gas can be obtained based on the laminar flow theory of the gas between the two flat plates [34].

(4) $$Re_{c}=\frac{2v_{c}\delta_{r}}{3\nu_{c}}$$

### 4.1. Radial Clearance Dimensions

**Figure 5:**
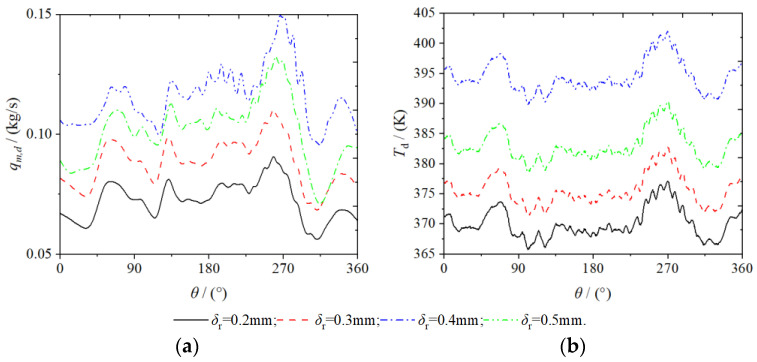
Mass flow and temperature (**a**) discharge port of mass flow (**b**) discharge port of temperature.

Figure 5 shows the variation in the outlet temperature and mass flow of the scroll expander for spindle rotation angle set at different radial clearances. The larger the radial gap clearance, the greater the amount of gas leak from the high-pressure chamber to the low-pressure chamber. This triggers the accumulation of gas in the low-pressure chamber, resulting in an increase of the outlet mass flow and temperature of the expander increase with the increase in radial clearance size. Owing to the radial clearance size increase in equal proportions, the trend in outlet mass flow and temperature was essentially the same.

The average temperature and mass flow at the outlet of the scroll expander and the volumetric efficiency of the expander at different radial clearances are presented in Table 3; where, $\bar{T}d$ and ${\bar{q}}_{m,d}$ are the average outlet temperature and mass flow, respectively, ${\bar{\varepsilon }}_{T}$ and $\varepsilon_{q,m}$ are the differences between the average outlet temperature and mass flow. respectively, and $\varepsilon_{\eta }$ is the difference in volumetric efficiency. Although the average mass flow and temperature at the outlet of the scroll expander increased with increase in the radial clearance, the volumetric efficiency decreased, which resulted in severe performance degradation of the scroll expander. As the radial clearance increases equi-proportionally, the law of change in the average outlet temperature and mass flow and volumetric efficiency do not change equi-proportionally. Compared with the set radial clearance ($\delta_{r}=$ 0.2 mm), the differences in the average mass flow at the outlet were 0.0083, 0.0302, and 0.0360 kg/s, respectively. Further, the differences in the average temperature at the outlet were 5.5505, 12.9512, and 24.4222 K, respectively. Finally, the differences in the volumetric efficiency were −0.6015, −3.1606, and −5.0521%, respectively.

**Figure 6:**
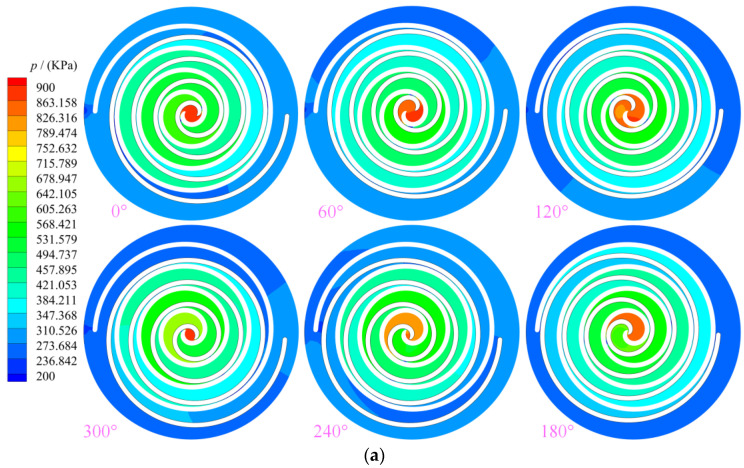

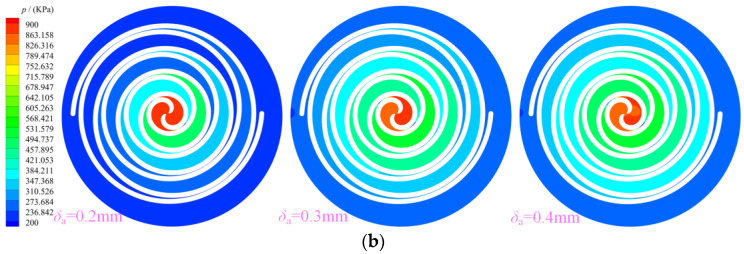
Pressure (**a**) Pressure (z=12.5 mm, $\delta_{r}=$ 0.5 mm) (**b**) Different gap of pressure (z= 12.5 mm, θ= 240°).

Figure 6 shows the distribution law of the pressure contour in the working chamber of the scroll expander. Owing to the pressure difference between adjacent working chambers, the clearances leak from the high-pressure chamber to the low-pressure chamber through the radial clearance, resulting in tangential leakage. Moreover, the higher the pressure difference between adjacent working chambers, the greater the amount of gas leakage. As shown in Figure 6a, with the continuous rotation of the orbiting scroll, the gas is subjected to different degrees of expansion as the suction, discharge, and back pressure chambers are always connected to the external environment. Thus, the uneven distribution of pressure in these working chambers is most obvious, and the pressure in the symmetrical working chamber of the same name in the expander also exhibits uneven distribution. When the spindle rotation angle is in the range of 60 to 120°, the asymmetrical arrangement of the inlet causes the pressure distribution in the suction chamber to become uneven. As shown in Figure 6b, when the spindle angle turns to 120°, the primary suction process of the scroll expander is not yet completed at this moment; thus, owing to the existence of the radial clearance, the gas in the suction chamber continuously flows into the expansion chamber, thereby resulting in the pressure distribution in the suction chamber becoming uneven. With decrease in the radial clearance in equal proportions, the pressure in the suction chamber gradually tended to be evenly distributed, thus allowing for a more complete expansion process of the gas.

**Figure 7:**
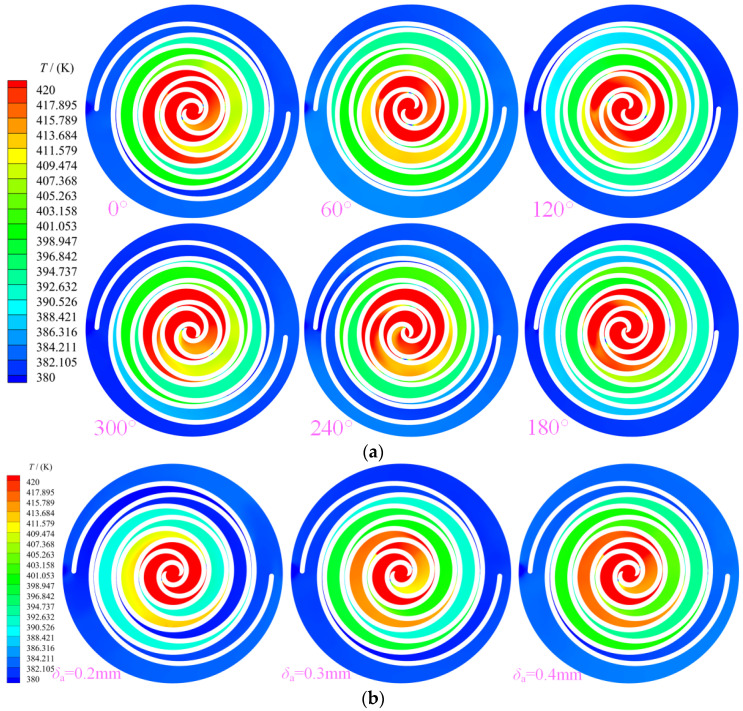
Temperature (**a**) Temperature (z=12.5 mm, $\delta_{r}=$ 0.5 mm) (**b**) Different gap of temperature (z= 12.5 mm, θ= 240°).

Figure 7 shows the temperature contour distribution law in the working chamber of the scroll expander. The tangential leakage flow is the main factor causing the non-uniform temperature distribution in the working chamber. As heat transfer was not considered in the three-dimensional unsteady state numerical simulation, the temperature of the gas in the working chamber was not affected by heat transfer. However, as shown in Figure 7a, the law of temperature distribution within the working chamber was inconsistent, even within the mutually symmetrical crescent-shaped working chamber of the same name. The temperature was only uniformly distributed in the suction chamber, whereas in all other working chambers an uneven distribution was observed, particularly in the expansion chamber. The distribution of temperature exhibits a gradual decrease from the suction chamber to the back-pressure chamber. The uneven temperature distribution in the working chamber can be primarily attributed to the tangential leakage flow disturbing the uniform distribution of the gas in the working chamber. As shown in Figure 7b, with increase in the radial gap size in equal-proportion, greater amounts of gas are released from the high-pressure chamber into the low-pressure chamber. Consequently, more heat is carried into the low-pressure chamber by the gas, thus causing the flow field distribution of the gas in the low-pressure chamber to be affected. In particular, the temperature in the second expansion chamber E_21_ will be significantly higher than that in the working chamber of the expander with other radial clearances. The larger the radial clearance, the greater the amount of heat carried by the gas and the higher the temperature of the gas accumulation in the low-pressure chamber. With further gas accumulates in the low-pressure chamber, the pressure in the chamber increases, which will cause the pressure in the chamber to create a certain obstruction to the leakage flow, thereby resulting uneven distribution of the leakage flow in the expansion chamber.

**Figure 8:**
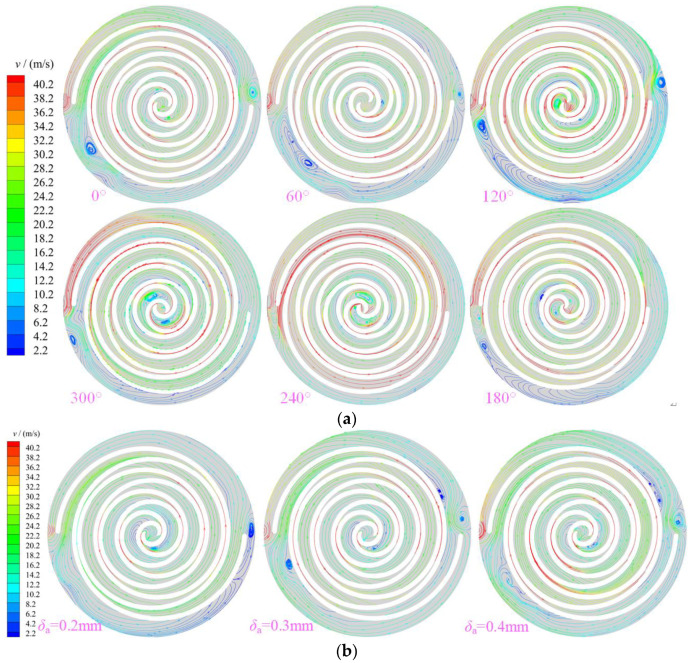
Stream traces (**a**) Velocity (z=12.5 mm, $\delta_{r}=$ 0.5 mm) (**b**) Different gap of velocity (z= 12.5 mm, θ= 240°).

Figure 8 shows the distribution of stream traces in the working chamber of the scroll expander. The maximum value of the velocity appeared at the clearance between the orbiting and fixed scroll teeth. Owing to the influence of the leaking gas, the distribution law of the velocity in different working chambers was also inconsistent. The velocity in the suction and back pressure chambers was higher than that in the expansion and discharge chambers, whereas that at the radial clearance was higher than that in other areas. Figure 8a shows the distribution law of stream traces in the working chamber of the scroll expander with the spindle rotation angle with radial clearance of 0.5 mm. For the scroll expander, in the closed crescent-shaped working chamber, the gas is disturbed by the orbiting scroll teeth and the mass exchange between adjacent working chambers, which causes the gas to form vortices of different scales and strengths in different working chambers. These vortices recur and dissipate with the rotation of the teeth of the orbiting scroll. During the flow of gas from the suction to the back-pressure chamber, the gas flow form is different in different working chambers. The main forms of gas flow are main flow, passage vortex, and secondary flow. The gas forms passage vortex and secondary flow in the expansion chamber owing to the combined effect of tangential leakage and main flows [18]. Figure 8b shows a contour of the stream-trace distribution of the gas in the working chamber at different radial clearances for a spindle rotation angle of 0°. The larger the radial gap size, the greater the amount of gas released from the high-pressure chamber into the low-pressure chamber. Consequently, the flow of gas in the working chamber of the expander is significantly disturbed, particularly in the E_12_ expansion chamber and the back-pressure chamber will form a different number, size, and strength of vortices. Further, the larger the radial clearance size, the greater the number and strength of vortices formed in the back-pressure chamber. As the radial clearance size increased in equal proportion, the maximum values of the velocity in the working chamber were all obtained at the radial gap. At P_1_, the velocities were 129.69, 150.699, 152.173, and 156.847 m/s, respectively. Moreover, at P_2_, the velocities were 131.399, 151.949, 155.46, and 161.544 m/s, respectively. In addition, the gas always flowed at subsonic speed in the working chamber.

**Figure 9:**
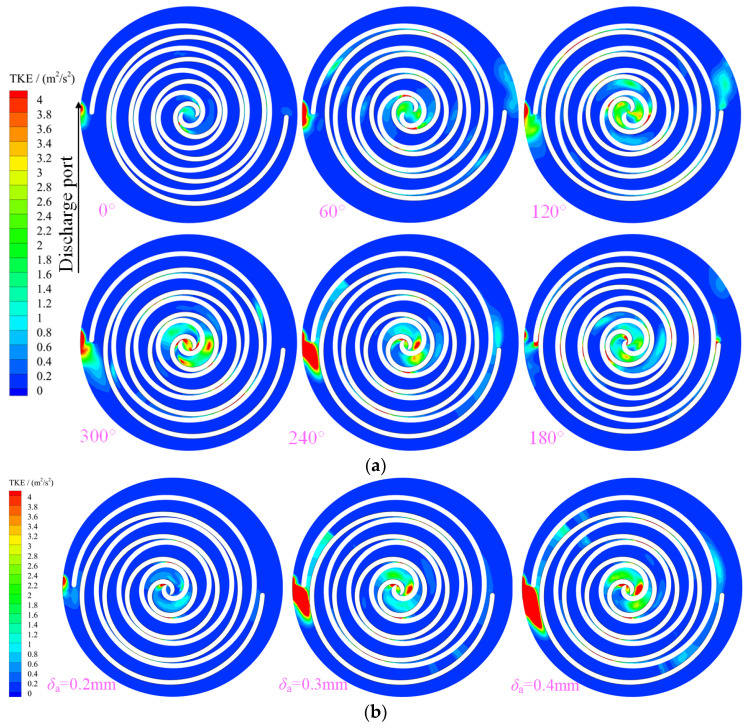
Turbulent kinetic energy (**a**) Turbulent kinetic energy (z= 12.5 mm, $\delta_{r}=$ 0.5 mm) (**b**) Different gap of Turbulent kinetic energy (z= 12.5 mm, θ= 240°).

Figure 9 shows the distribution of turbulent kinetic energy (TKE) in the working chamber of a scroll expander. The TKE is mainly influenced by the velocity and Reynolds number in the working chamber. The higher the velocity, the higher the turbulent kinetic energy. Further, the maximum values of TKE in the working chamber were located in the suction, first expansion, and back-pressure chambers in the range 240 to 300°. The TKE in the first expansion and suction chambers increased with the angle of rotation from 180 to 300°. At a spindle rotation angle of 300°, the maximum value of TKE in the first expansion chamber E_12_ was obtained near the middle of the working chamber. Further, at a spindle rotation angle of 240°, a sudden increase in TKE in the back-pressure chamber at BAC_1, near the outlet, was caused by the structure of the outlet pipe. Figure 9b shows the distribution law of TKE in the working chamber of the scroll expander at different radial clearances for a spindle rotation angle of 240°. With decrease in the radial gap size in equal-proportion, the TKE in the suction chamber, first expansion chamber, radial gap, and back-pressure chamber of the scroll expander also decreased.

**Figure 10:**
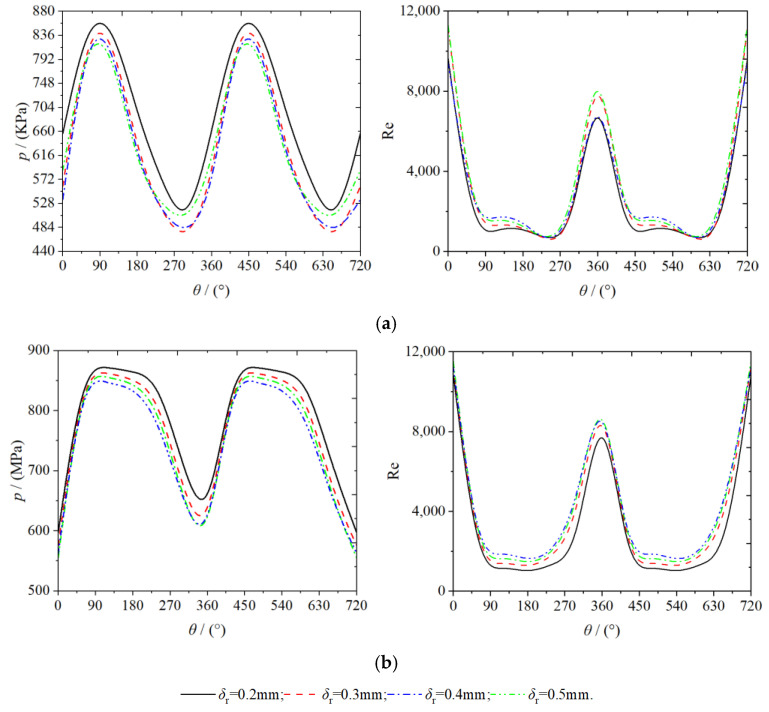
Monitoring point data (**a**) P1 (**b**) P2.

Figure 10 shows the variation law of pressure and Reynolds number with spindle rotation angle at the position of the monitoring point. As evident from Figure 6, Figure 7, Figure 8 and Figure 9, the distribution law of the physical properties of the gases were not consistent within the working chambers of the scroll expander. The same behavior was observed even within the mutually symmetrical working chambers of the same name. In the working chamber, the pressure at the monitoring point tended to increase and then decrease, whereas the Reynolds number tended to decrease and then increase, with continued rotation for the teeth of the orbiting scroll. This indicates that the physical properties of the gas do not change uniformly at the gap between the teeth. The minimum value of pressure and the maximum value of Reynolds number occur at the point of engagement of the fixed and orbiting scroll teeth, respectively. Owing to the loss of suction pressure in the suction process of the scroll expander, the gas pressure in the first expansion chamber were all lower than the set suction pressure ($p_{s}$ = 900 KPa). Moreover, as a part of the E_12_ expansion chamber was connected to the inlet pipe, the average pressure in the chamber was higher than in the E_11_ expansion chamber. In the working chamber the Reynolds number was mainly influenced by the velocity; therefore, the trend of the Reynolds number and the velocity were the same. When the spindle rotation angle is in the range of 0 to 35° and 309 to 360°, the flow of the gas in this range can be considered turbulent because the gas Reynolds number value is greater than 4000, whereas in the other moments of motion, the gas exhibits a laminar flow [17]. At monitoring points P_1_ and P_2_, both pressure and Reynolds number decreased with increasing radial clearance. As the radial clearance size increased proportionally, the average pressures at P_1_ were 683.8458, 643.5845, 624.9455, and 607.2163 KPa respectively. Further, the average pressures at P_2_ were 773.7025, 760.1604, 748.6414, and 743.1457 Kpa, respectively. In addition, the average Reynolds numbers at P_1_ were 2894.1342, 3339.7610, 3561.4566, and 3757.1245, respectively; and those at P_2_ were 4622.1492, 4461.2918, 4328.1109, and 4015.6971, respectively.

### 4.2. Rotational Speed

**Figure 11:**
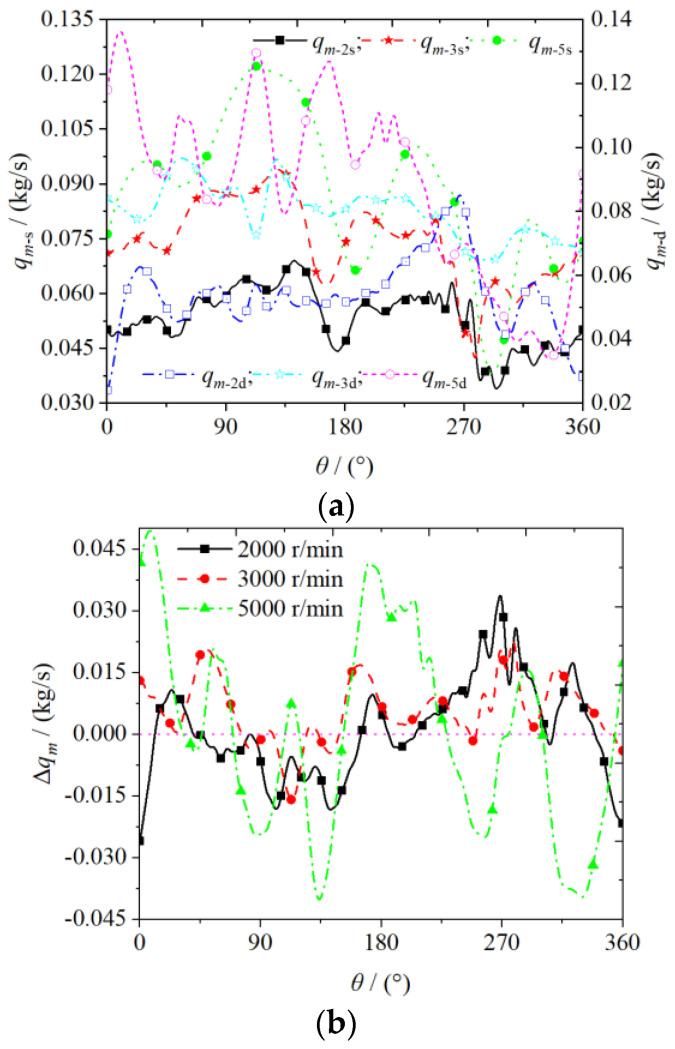
Mass flow (**a**) Suction and discharge port mass flow (**b**) Suction and discharge port mass flow of difference.

Figure 11 shows the variation of the mass flow at the inlet and outlet of the scroll expander with the spindle rotation angle set at different rotational speeds. The mass flow at the inlet and outlet of the scroll expander increased with increase in the rotational speed, whereas the tangential leakage decreased. As shown in Figure 11a, the higher the rotational speed of the scroll expander, the more gas inhaled from the outside. Consequently, the mass flow at the inlet and outlet of the expander increases accordingly. In the range of 270–360° of spindle rotation angle, owing to the possibility of the orbiting scroll causing different degrees of blockage to the scroll expander inlet (resulting in reduced inlet area), such that the expander inlet and outlet mass flow are reduced. As shown in Figure 11b, the difference in mass flow between the inlet and outlet of the scroll expander varies with the spindle rotation angle at different rotational speeds. The presence of the radial gap causes the gas to build up in the low-pressure chamber, thereby resulting in the outlet mass flow of the scroll expander slightly higher than the inlet mass flow. As the rotational speed increases, the average mass flow differences between the inlet and outlet were $112\times 10^{-6}$, $35\times 10^{-6}$, and $10^{-6}$ kg/s, respectively. As the tangential leakage does not occur at high rotational speeds, the gas can expand sufficiently in the working chamber such that the difference between the inlet and outlet mass flow gradually tends to 0. Therefore, increasing the speed reduces the amount of internal leakage generated between adjacent working chambers of the scroll expander.

Table 4 presents the effect of rotational speed on the average outlet temperature and mass flow, as well as the volumetric efficiency of the scroll expander. The higher the rotational speed of the scroll expander, the lesser the amount of tangential leakage generated between adjacent working chambers. In addition, the amount of tangential leakage from the high-pressure chamber into the low-pressure chamber is also reduced. Consequently, the average mass flow at the expander outlet and volumetric efficiency increased whereas the average temperature at the outlet decreased. The increase in volumetric efficiency increases the output power of the scroll expander, thereby improving the work efficiency of the entire Micro-scale CAES system. Moreover, at a constant radial clearance, increasing the rotational speed of the expander facilitates the complete expansion of the gas in the working chamber, which reduces the average outlet temperature. When the rotational speed was increased from 2000 to 5000 r/min, the average outlet mass flow and volumetric efficiency increased by 0.0613 kg/s and 8.7565% respectively, whereas the average outlet temperature decreased by 11.8683 K. During the actual operation of a scroll expander, the average outlet temperature increases with rotational speed owing to frictional heat generation, inadequate cooling and heat transfer.

**Figure 12:**
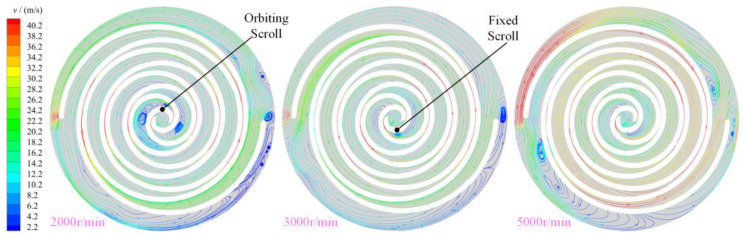
Stream traces (z= 12.5 mm, θ= 360°).

Figure 12 shows the distribution law of the stream traces in the working chamber of the scroll expander at different rotational speeds. With increase in the rotational speed, the velocity of the gas in the working chamber also increases, particularly in the radial clearance and the back-pressure chamber BAC_2. As shown in Figure 8, vortices of different intensities and sizes were generated in the first expansion chamber owing to the tangential leakage flow. However, with increase in the rotational speed of the scroll expander, the tangential leakage decreases. Consequently, the intensity of the vortices formed by the gas in the first expansion chamber decreased at high rotational speeds; at a rotational speed of 5000 r/min, the vortices even disappear. Thus, the size and strength of the vortices in the first expansion chamber were influenced by the rotational speed, the lower the rotational speed the wider the size range and the greater the strength of the vortices. At low rotational speed, at least five vortices of different sizes and strengths appeared in the back pressure and near the end of the orbiting scroll teeth, respectively. The formation of vortices in the back-pressure chamber was influenced by the combination of the main and tangential leakage flows, rotation of the orbiting scroll, and gas flow in the outlet pipe. At low rotational speed the gas flow in the back-pressure chamber was the most complex and the number of vortices in the chamber was the highest owing to the high number of tangential leaks generated. Further, at high rotational speed, the size and strength of the vortex in the back-pressure chamber increased because of the influence of the movement of the orbiting scroll and the gas flow in the outlet pipe. The flow direction and flow law of the fluid changed when close to the end of the orbiting scroll tooth. The lower the rotational speed, the further away from the end of the teeth the initial position of the change in gas flow.

**Figure 13:**
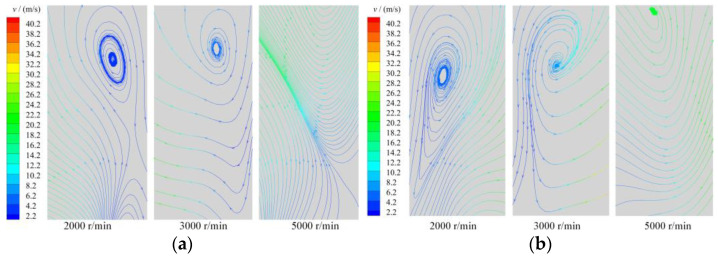
Streamtraces (*z* = 12.5mm, θ= 360°) (**a**) E11 (**b**) E12.

Figure 13 shows the streamline distribution of the gas in the working chamber of the scroll expander along the direction of the tooth height. The distribution of the gas was also uneven in the direction of the tooth height along the scroll teeth. The lower the rotational speed, the more complex the gas flow law in the working chamber. With increase in the rotational speed, the strength, size, and even disappearance of the vortex formed by the gas flow in the first expansion chamber were reduced. In the E_11_ and E_12_ expansion chambers, the flow law and direction of gas were also inconsistent. Specifically, the gas in E_11_ expansion chamber flowed from the bottom to the top, whereas that in E_12_ flowed from the top to the bottom; the higher the rotational speed, the simpler the gas flow law along the tooth-height direction.

### 4.3. Different Inlet Pressures and Temperatures

**Figure 14:**
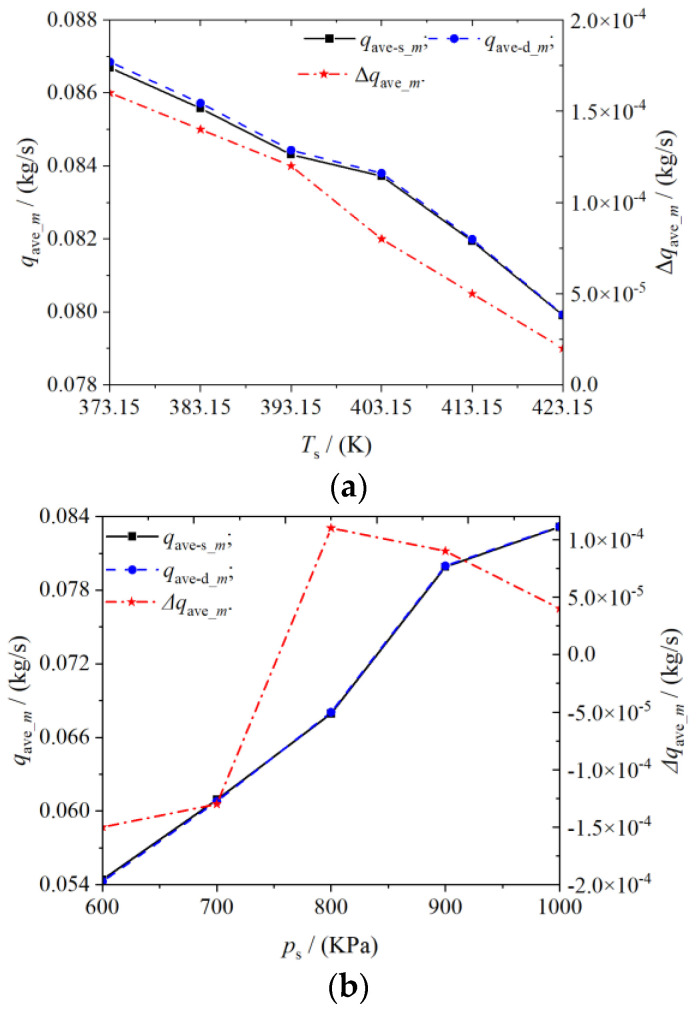
Average inlet and outlet mass flow (**a**) Different suction port pressure (**b**) Different suction port temperature.

Figure 14 shows the variation of the average mass flow of the inlet and outlet of the scroll expander with the inlet pressure and temperature. The variation of pressure and temperature in the working chamber can also affect the tangential leakage. As shown in Figure 14a, the inlet and outlet mass flow of a scroll expander increased with increase in the inlet pressure, whereas the difference between the inlet and outlet mass flow decreased. At a constant radial clearance size, increasing the inlet pressure increases the amount of gas flowing from the expander inlet into the suction chamber, resulting in a consequent increase in the outlet mass flow. The higher the pressure in the working chamber, the greater the differential pressure between adjacent working chambers, which reduces the tangential leakage. As shown in Figure 14b, increasing the inlet temperature of the expander causes the gas density to decrease. Therefore, the inlet and outlet mass flows also decrease. However, the difference between the inlet and outlet mass flow increases with the increase of inlet temperature.

As the effect of temperature on the deformation of the orbiting and fixed scroll was not considered in the numerical simulation of the scroll expander in this study, the effect of temperature on the difference between the inlet and outlet mass flow was also small. In the actual operation of the scroll expander, with increase in the inlet temperature, certain deformation of the fixed and orbiting scroll teeth is observed. Consequently, the radial gap size decreases, which further reduces the tangential leakage through the radial gap. Thus, the actual difference between the inlet and outlet mass flow will be lower than the numerical simulation of the difference.

## 5. Conclusions

This study conducted the three-dimensional unsteady numerical simulation of oil-free air scroll expander with radial clearance. The generation mechanism and change rule of tangential leakage flow as well as the distribution rule of non-uniform flow field in the working chamber, were emphatically studied. In addition, the variation laws of the mass flow at the inlet and outlet of the expander, pressure, temperature, and velocity contour in the working chamber under different operating conditions were also studied. The main conclusions of this study are as follows:

(1) Through the radial clearance, there is a mass exchange between adjacent working chambers of the scroll expander. The leaking gas affected the distribution law of the gas in the low-pressure chamber, which resulted in a non-uniform distribution of the pressure, temperature, and velocity of the gas in the working chamber. In addition, a higher physical quantity of gas was observed in the expansion chamber E*_i_*_1_ chamber than in E*_i_*_2_. The distribution law of these physical quantities is as follows: from the suction chamber to the back-pressure chamber, the distribution law follows a trend of high to low.

(2) With an equal-proportional increase in radial clearance size, the average outlet temperature increased by 1.5, 3.5, and 6.6%, respectively, compared to the initial radial clearance ($\delta_{r}=$ 0.2 mm), and the average mass flow at the outlet increased by 10.3880, 37.7972, and 45.0563%, respectively; whereas, the volumetric efficiency decreased by 0.7492, 3.9368, and 6.2928%, respectively. Further, increasing the radial clearance size reduced the volumetric and working efficiencies of the scroll expander. The tangential leakage through the radial clearance severely disturbed the flow field distribution of the gas in the working chamber of the scroll expander. Further, the gas distribution in the first expansion chamber was most affected with the equal-proportion increase of the radial clearance. Thus, the tangential leakage flow exerts a greater influence on gas temperature, velocity, and turbulent kinetic energy, but less influence on pressure.

(3) With increase in the rotational speed of the scroll expander, the tangential leakage from the high-pressure chamber into the low-pressure chamber is reduced, with all other initial calculation conditions being constant. When the rotational speed of the scroll expander decreased from 5000 to 2000 r/min, the average outlet temperature of the scroll expander decreased by 3.1291%, and the mass flow and volumetric efficiency increased by 112.4771% and 11.3822%, respectively. Thus, the higher the rotational speed, the less the corresponding tangential leakage, and the more stable the gas flow in the working chamber.

(4) Based on the premise that the outlet pressure remains unchanged, with the increase in the suction pressure of the scroll expander, the pressure difference between adjacent working chambers also increased, thus reducing the tangential leakage. Further, increasing the inlet temperature also reduced the tangential leakage in the working chamber of the scroll expander. With the equal-proportion increase of inlet pressure and temperature, the average mass flow difference between inlet and outlet decreased from $15\times 10^{-5}$ to $4\times 10^{-5}$ kg/s, and from $16\times 10^{-5}$ to $2\times 10^{-5}$ kg/s, respectively.

## Figures and Tables

**Table 1:** Grid independence verification.

Number of Grids	Volumetric Efficiency (%)	Calculation Time (Hours)
1,169,982	80.0591	10
2,014,142	80.2236	16
2,858,032	80.2832	30
3,702,462	80.2845	42

**Table 2:** Simulation and experimental results.

Method	Volumetric Efficiency (%)	Isentropic Efficiency (%)
Simulation value	80.2832	41.3265
Experimental value	89.1456	47.5254

**Table 3:** Radial clearances impact.

$\delta_{a}\;(\mathrm{mm})$	${\bar{T}}_{d}\;(K)$	$\varepsilon_{T}\;(K)$	${\bar{q}}_{m,d}\;(\mathrm{kg}/s)$	$\varepsilon_{qm}\;(\mathrm{kg}/s)$	$\eta_{v}\;(\%)$	$\varepsilon_{\eta }\;(\%)$
0.2	370.0328	-	0.0799	-	80.2832	-
0.3	375.5833	5.5505	0.0882	0.0083	79.6817	−0.6015
0.4	382.9840	7.4007	0.1101	0.0219	77.1226	−2.5591
0.5	394.4550	11.4710	0.1159	0.0058	75.2311	−1.8915

**Table 4:** Rotational speed impact.

*n* (r/min)	${\bar{T}}_{d}$ (K)	$\varepsilon_{T}$ (K)	${\bar{q}}_{m,d}$ (kg/s)	$\varepsilon_{qm}$ (kg/s)	$\eta_{v}$ (%)	$\varepsilon_{\eta }$ (%)
2000	379.2842	9.2514	0.0545	−0.0254	76.9314	−3.3518
3000	370.0328	-	0.0799	-	80.2832	-
5000	367.4159	−2.6169	0.1158	0.0359	85.6879	5.4047

## Data Availability

Not applicable.

## Nomenclature

$r_{b}$	radius of base circle, mm
*h*	scroll tooth height, mm
Suc	Suction chamber
Bac	Backpressure chamber
Dis	Discharge chamber
E	Expression chamber
P1 and P2	Monitoring points
D	Source term
s and d	Suction and discharge port
$p_{s}$ and $p_{d}$	Suction and discharge port pressures, KPa
n	Rotational speed, r/min
Y+	Y Plus
$T_{s}$	Suction port temperature, K
$m_{sim}$	Simulation suction port mass flow, kg/s
$V_{s}$	Suction volume, $m^{3}$
$v_{c}$	Velocity at the monitoring point, m/s
$T_{d}$	Discharge port temperature, K
${\bar{T}}_{d}$	Average discharge port temperature, K
$q_{m,s}$ and $q_{m,d}$	Suction and discharge port mass flow, kg/s
$q_{m,(2/3/5)s}$ and $q_{m,(2/3/5)d}$	Suction and discharge port mass flow rates at different speeds, kg/s
$q_{m,d\_\mathrm{ave}}$	Discharge port average mass flow, kg/s
Greek symbols	
α	Involute generation angle, rad
ψ	Solution variable
μ,ν,χ, and ζ	General variables
$\eta_{v}$	Volumetric efficiency
$p_{s}$	Density of the inlet, ${\mathrm{kg}/m}^{3}$
$\delta_{r}$	Radial clearance, mm
$\nu_{c}$	Gas viscosity gas, kg/(m⋅s)
θ	Orbiting angle, °
$\varepsilon_{T}$	Differences between the average outlet temperature, K
$\varepsilon_{qm}$	differences between the average outlet mass flow, kg/s
$\varepsilon_{\eta }$	difference in volumetric efficiency
